# Biomimetic Silk Nanoparticle Manufacture: Calcium Ion-Mediated Assembly

**DOI:** 10.1021/acsbiomaterials.4c02175

**Published:** 2025-01-30

**Authors:** Napaporn Roamcharern, Saphia A. L. Matthew, Daniel J. Brady, John A. Parkinson, Zahra Rattray, F. Philipp Seib

**Affiliations:** a Strathclyde Institute of Pharmacy and Biomedical Sciences, University of Strathclyde, 161 Cathedral St., Glasgow G4 0RE,Scotland,U.K.; b Branch Bioresources, Fraunhofer Institute for Molecular Biology and Applied Ecology, Ohlebergsweg 12, Giessen 35392, Germany; c Department of Pure and Applied Chemistry, University of Strathclyde, 295 Cathedral Street, Glasgow G1 1XL, Scotland,U.K.; d Institute of Pharmacy, Department of Pharmaceutics and Biopharmaceutics, Friedrich Schiller University Jena, Lessingstr. 8, Jena 07743, Germany

**Keywords:** *Bombyx mori*, silk fibroin, antisolvent precipitation, desolvation, metal ion, nanomedicine

## Abstract

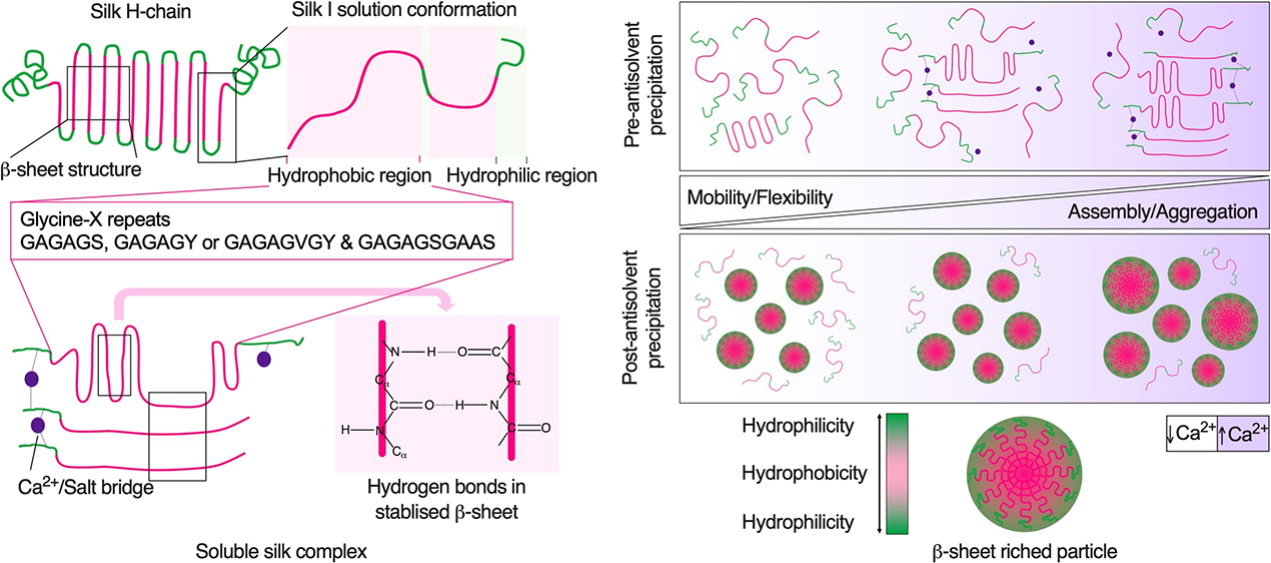

Silk has emerged as an interesting candidate among protein-based nanocarriers due to its favorable properties, including biocompatibility and a broad spectrum of processing options to tune particle critical quality attributes. The silk protein conformation during storage in the middle silk gland of the silkworm is modulated by various factors, including the most abundant metallic ion, calcium ion (Ca^2+^). Here, we report spiking of liquid silk with calcium ions to modulate the silk nanoparticle size. Conformational and structural analyses of silk demonstrated Ca^2+^-induced silk assemblies that resulted in a liquid crystalline-like state, with the subsequent generation of β-sheet-enriched silk nanoparticles. Thioflavin T studies demonstrated that Ca^2+^ effectively induces self-assembly and conformation changes that also increased model drug loading. Ca^2+^ incorporation in the biopolymer feed significantly increased the nanoparticle production yield from 16 to 89%, while simultaneously enabling Ca^2+^ concentration-dependent particle-size tuning with a narrow polydispersity index and altered zeta potential. The resulting silk nanoparticles displayed high biocompatibility in macrophages with baseline levels of cytotoxicity and cellular inflammation. Our strategy for manufacturing biomimetic silk nanoparticles enabled overall tuning of particle size and improved yields—features that are critical for particle-based nanomedicines.

## Introduction

Silk has emerged as a promising biopolymer for expanding the repertoire of bioinspired drug-delivery carriers that presently include collagen,^1^ chitosan,^2^ cellulose,^3^ among others. Silk serves as a useful benchmark for biomedical applications by virtue of its favorable biodegradability, biocompatibility, and protecting payload from degradation.^4−6^ Silk biomolecular characteristics are fundamentally governed by its primary amino acid sequence and orchestrated assembly, which results in an overall hierarchical structure. Controlling this hierarchal structure broadens the range of silk applications, including silk-based drug-delivery applications.^7^

At the molecular level, *Bombyx mori* silk is composed of a high-molecular-weight heavy chain (H-chain, 390 kDa), a low-molecular-weight light chain (L-chain, 26 kDa), and the glycoprotein fibrohexamerin (formally P25) (30 kDa). The H-chain possesses amorphous regions (β-turns, α-helixes, and random coils) and a crystalline region (β-sheet), which account for 33, and 66% of the total sequence, respectively.^8^ Nonrepeating amino acid residues are capping the silk sequence at the N- and C-termini of the H-chain. The hydrophobic block of the H-chain is a repetitive sequence of glycine-X (GX) repeats, where X is alanine (A), serine (S), or tyrosine (Y) and accounts for 65, 23, and 9% of the sequence, respectively. These GX repeats are ordered by three key sequences: (i) GAGAGS, (ii) GAGAGY or GAGAGVGY, and (iii) GAGAGSGAAS. The H-chain is composed of 12 hydrophobic blocks linked together by 11 hydrophilic blocks of amorphous regions, each consisting of 31 amino acids. Each repetitive sequence of the H-chain hydrophobic blocks governs different structural characteristics. The GAGAGS sequence contributes to the bulk of the crystalline regions and is typically located at the beginning of each motif, whereas the GAGAGY or GAGAGVGY sequence forms semicrystalline regions, and GAGAGSGAAS acts as a sheet-breaking motif.^7,9−11^

In the silk gland, the structure of the silk protein is in part modulated by ions, which guide structural transformation from the liquid storage form (amorphous silk I) to the “spinning ready” form. Calcium ion (Ca^2+^) levels decrease from the posterior to the anterior segments of the middle silk gland, from approximately 3000 to 500 mg/g dry luminal content weight.^12^ In the presence of Ca^2+^, a salt bridge is formed between Ca^2+^ and the hydrophilic groups of the silk molecule. These salt bridges stabilize the silk conformation during storage, thereby increasing the silk solution viscosity to present a gel-like liquid silk state.^12,13^

Investigations into the conformational and interactional dynamic alterations of silk are now increasing our knowledge of the control of silk structure before and after silk fiber spinning.^14,15^ Various analytical techniques, such as nuclear magnetic resonance (NMR) and Fourier transform infrared (FTIR), are routinely deployed for the characterization of silk.^13−18^ Solid- and solution-state NMR has been used to classify the silk structure and the conformational transitions that dictate the physicochemical properties of each unique repeated sequence.^8,14^ This information can be harnessed to engineer silks with favorable characteristics. For example, FTIR-based studies on the secondary structure and conformational changes occurring in Ca^2+^-mixed *Antheraea pernyi* silk fibroin showed differences in the absorption bands (amide I–VI; 1655, 1545, 892, and 1270 cm^–1^, respectively) over time associated with the β-sheet degree.^19^ These changes have important consequences in the fabrication of silk drug-delivery systems, as they affect the solubility and, therefore, the likelihood of precipitation of silk to form silk nanoparticles.

Antisolvent nanoprecipitation is one of the least energy-intensive laboratory-scale methods, as it requires no complex procedures or expensive apparatus, and relies simply on the spontaneous self-assembly of proteins to form nanoparticles (25–180 nm).^20^ Therefore, an increasing number of studies are adopting this approach to manufacture silk nanoparticles.^6,21^ For example, a semibatch procedure involving the drop-by-drop addition of reverse-engineered silk solution into isopropanol induced structural changes in which the low solubility of the hydrophilic blocks of the H-chain resulted in the self-assembly of β-sheet-rich silk nanoparticles (∼100 nm).^20^ Subsequent method refinements^22,23^ and the use of liquid silk stocks with different molecular-weight characteristics^23^ demonstrated the ability to tune nanoparticle properties. Despite this, the implementation of biomimetic principles to orchestrate silk nanoparticle self-assembly and improve yields has not been studied to date. The aim of the present study was to evaluate the impact of physiologically relevant calcium ion concentrations on the critical quality attributes of silk nanoparticles manufactured by alcohol-mediated nanoprecipitation in a semibatch format while titrating the calcium ion concentration. We hypothesized that this biomimetic approach could fine-tune the critical quality attributes of silk nanoparticles to achieve a predetermined particle size range and a greater production yield of silk-based nanocarriers.

## Experimental Section

### Aqueous Silk Preparation and Silk Nanoparticle Synthesis

The procedures used to manufacture silk nanoparticles have been described elsewhere.^20^Figure S1 shows the workflow used for silk nanoparticle and aqueous silk fibroin preparation (referred to as silk in the manuscript). Briefly, *B. mori* silk cocoons were chopped into 5 × 5 mm pieces. Next, 5 g of cocoon pieces were degummed by boiling in 2 L of 0.02 M Na_2_CO_3_ for 1 h under gentle stirring. Degummed silk was rinsed with 200 mL of deionized water before immersion in 1 L of DI water for 20 min. The washing step was performed twice more, squeezing the fibers to remove excess liquid and then drying them overnight at ambient temperature in a fume hood.

The dry degummed silk was dissolved in a warm solution of 9.3 M LiBr with a silk:LiBr ratio of 1 g to 4 mL and then incubated at 60 °C for 4 h. The resulting silk was dialyzed against deionized water (Slide-A-Lyzer 3.5K Dialysis Cassette G2, Thermo Scientific, Rockford, IL, USA) for 48 h, centrifuged three times at 2885 × *g* for 40 min at 4 °C (PK 121R Centrifuge, rotor T515, ACL International Srl, Milan, Italy), and stored at 4 °C until further analysis. A 500 mL aliquot of silk was dried at 60 °C for 48 h for % w/v calculations.

Silk nanoparticles were manufactured using a drop-by-drop approach in a semibatch format. First, silk was mixed with a CaCl_2_ solution to yield 6 mL of 3% w/v silk containing a final mass ratio of Ca^2+^:silk at 0.7 and 11.5 mg/g. The mixture was then added dropwise to isopropanol at a syringe pump speed of 1 mL/min, a height of 7.5 cm, and a stirring speed of 400 rpm. The resulting silk nanoparticles were centrifuged at 48,400 × *g* at 4 °C for 2 h, followed by sonication in deionized water. The centrifugation and sonication cycles were repeated twice more to ensure the complete removal of contaminants. Silk nanoparticles were produced in triplicate using different silk batches (*n* = 3).

### Fourier Transform Infrared Spectroscopy

The secondary structure of silk and the silk nanoparticles was determined by Fourier transform infrared (FTIR) spectroscopy. All samples were prefrozen at −20 °C overnight prior to lyophilization at −10 °C and 0.140 mbar for 24 h. Freeze-dried silk and 70% ethanol-treated silk films were prepared as a reference for silk I and II crystalline structures, respectively. FTIR measurements were collected over the wavenumber range of 400–4000 cm^–1^ with a 4 cm^–1^ resolution (ATR-equipped TENSOR II FTIR spectrometer, Bruker Optik GmbH, Ettlingen, Germany). The scanning time was set at 32 and 128 scans for the background and sample channel scans, respectively. The secondary structure content was deconvoluted and calculated from the FTIR absorbance spectra in the region of amide I. The following wavenumbers were referenced for the identification of each secondary structure component as described previously with minor modification:^20^ 1697–1710 cm^–1^ for the antiparallel amyloid β-sheet structure, 1660–1690 cm^–1^ for the β-turn structure,^32^ 1638–1655 cm^–1^ for the random coil structure, 1620–1635 cm^–1^ for the native β-sheet structure, and 1605–1625 cm^–1^ for the intermolecular β-sheet structure. Second-derivative amide I spectra (1600–1700 cm^–1^) were used in correlation coefficient (*R*) calculations versus the air-dried silk film, as described previously.^20^

### Nuclear Magnetic Resonance Spectroscopy

For selected studies, *B. mori* larvae were reared on artificial diet (CREA, Sericulture Laboratory of Padua, Italy) at 25 ± 1 °C and 75% relative humidity. Modified isotope-labeled silk cocoons were produced by incorporating 50 mg of d-glucose-^13^C_6_ (Cortec, Les Ulis, France) per 250 mg of dry artificial diet, fed twice daily over days 4–6 of the fifth instar, and allowed to spin their cocoons.

The silk material was prepared as described above using unmodified silk cocoons and modified isotope-labeled silk cocoons. The 500 mL of Ca^2+^-mixed silk solution was prepared by mixing unmodified and modified silk at a 1:1.5 protein mass ratio, yielding a final 3% w/v protein concentration supplemented with 0.7 and 11.5 mg Ca^2+^ per 1 g silk.

The impact of Ca^2+^ on the NMR spectroscopy response of silk samples was determined using a Bruker 600 MHz AVANCE II^+^ NMR spectrometer operating at 600.13 MHz for proton data observation and equipped with BBO-z or TBI-z probe heads depending on the application. All samples were equilibrated at a probe head temperature of 298 K. 1D ^1^H NMR spectra were typically acquired over a frequency width of 12 kHz (20 ppm) as 16 K data points (acquisition time = 681.5 ms) for each of 64 transients and centered at a transmitter offset frequency equivalent to δ^1^H = 4.698 ppm. Data were acquired using a Watergate pulse sequence combined with soft presaturation to eliminate the solvent resonance. 2D [^1^H, ^15^N] heteronuclear single quantum coherence (HSQC) NMR data were acquired over ω_2_ and ω_1_ frequency widths of 6 kHz (10 ppm) and 2.4 kHz (40 ppm) and centered at frequency offsets equivalent to δ^1^H = 4.698 ppm and δ^15^N = 120 ppm for direct ^1^H and indirect ^15^N resonance detection, respectively. The Bruker pulse program hsqcetf3gpsi was used to acquire NMR data as 2048 data points (acquisition time = 170 ms) with 16 transients for each of 128 *t*_1_ increments by using a traditional echo–antiecho data acquisition scheme. All data were processed using TopSpin version 4.0.5 (Bruker Biospin).

### Thioflavin T Fluorescence Measurement of Aqueous Silk Fibroin and Silk Nanoparticles

Thioflavin T was used to investigate the structural changes of aqueous silk fibroin in the presence of Ca^2+^. Briefly, 25 μL of 3% w/v silk containing Ca^2+^ (0.7 and 11.5 mg/1 g silk) was incubated with an equal volume of 100 μM thioflavin T in deionized water and 50 μL of deionized water. After a 10 min incubation at ambient temperature, the bound thioflavin T fluorescence intensity was read (excitation = 440 nm, emission = 475 nm) (Polarstar Omega, BMG Labtech, Ortenberg, Germany). The 60% v/v isopropanol served as a β-sheet-rich (silk II) positive control.

For the model drug-loading studies, thioflavin T served as the payload. The thioflavin T-loaded silk nanoparticles were manufactured as described above by supplementing the liquid silk mixture with 100 μL of 1000 μM thioflavin T. The thioflavin T-loaded silk nanoparticles were collected and characterized using the nanoparticle-tracking analysis (NTA 3.4 Build 3.4.003, Malvern Panalytical Ltd., Worcestershire, UK). Briefly, the 1 mg/mL suspension of thioflavin T-loaded silk nanoparticles was 500- to 5000-fold diluted in 0.2 μm filtered Milli-Q water. The particle size distribution, particle concentration, and fluorescence intensity were measured with the following capture and analysis settings: a sCMOS camera, Blue 488 laser, 500 nm long pass filter, syringe pump speed at 50, and viscosity at 1.0000 cP.

### Production Yield Determination

The production yield was calculated as detailed previously.^20^

### Size and Zeta Potential Measurements

Particle size and surface net charge were determined using dynamic light scattering and electrophoretic light scattering (Zetasizer Nano-ZS Malvern Instrument, Worcestershire, UK). Size analysis was performed using a 1:25 ratio (v/v) of 1 mg/mL silk nanoparticles to deionized water with the following settings: measurement temperature, 25 °C; count rate, 203.6 kcps; measurement duration, 70 s; measurement position, 3 mm. The zeta potential was measured using a 1:20 ratio (v/v) of 5 mg/mL silk nanoparticles to deionized water with the following settings: temperature, 25 °C; count rate, 161.5 kcps; zeta run, 12; measurement position, 2 mm.

### Morphology Analysis

Silk nanoparticle suspensions were sonicated twice at 30 amplitude for 30 min and prepared at 1 mg/mL in deionized water. The silk nanoparticle suspension (20 μL) was dropped onto a 5 × 5 mm silicon wafer chip (Ted Pella, Inc., CA, USA), dried at room temperature for 24 h, and gold sputter-coated from a 35 mm height for 40 s at 0.08 mb and 30 mA (Agar Scientific Manual Sputter Coater, Agar Scientific Ltd., Essex, UK). The silk nanoparticles were imaged by field emission scanning electron microscopy at 10,000-fold, 20,000-fold, and 60,000-fold magnifications at 5 kV (Hitachi SU6600, Hitachi High-Tech Europe GmbH, Krefeld, Germany).

### Cytotoxicity and Inflammation Measurements

RAW 264.7 murine macrophages were purchased from ATCC (product number TIB-71, ATCC, UK). The cells were cultured in DMEM supplemented with 10% FBS, 50 U/mL penicillin, and 50 mg/mL streptomycin. Seeded cells were processed at 80–90% cell confluency by scraping, centrifuging at 380 *× g* for 4 min, and replating at 15,000 cells/cm^2^. After 24 h, the cells were dosed with fresh complete medium containing 31.25–500 μg/mL silk nanoparticles and incubated in a 5% CO_2_ incubator at 37 °C for 48 h. Viability assays were then performed by exposing the cells to 20 μL of 5 mg/mL 3-[4,5-dimethylthiazol-2-yl]-2,5 diphenyl tetrazolium bromide solution for 4 h and recording absorbance at 570 nm (Multiskan Ascent V1.24, Thermo Fisher Scientific Inc., MA, USA). Cell viability was expressed as a percentage relative to the untreated control cells.

Endogenous nitrite (NO_2_^–^), tumor necrosis factor alpha (TNF-α), and the cytokine profile were measured as markers of cell responses to silk nanoparticle exposure. Cells treated with complete medium containing 200 ng/mL lipopolysaccharide served as an inflammatory-activated control. The tested concentration of silk nanoparticles was 500 mg/mL. Following 24 h incubation, the medium was collected and centrifuged at 21,630 *× g* for 5 min (VWR Micro Star 30 centrifuge, VWR International, LLC., Leicestershire, UK). The supernatant was transferred to a low-binding protein microtube and stored at −80 °C for further analysis.

Endogenous NO_2_^–^ levels in the supernatants were quantified using a Total Nitric Oxide and Nitrate/Nitrite Parameter Assay Kit (R&D Systems, Minneapolis, MN, USA), TNF-α levels were assayed using a Mouse TNF-alpha Quantikine ELISA Kit (R&D Systems, Minneapolis, MN, USA), and semiquantitative measurements of cytokines and chemokines were performed using the Proteome Profiler Array (Mouse Cytokine Array Panel A, R&D Systems, Minneapolis, MN, USA).

### Analysis Software and Statistical Analyses

All data were collated using Microsoft Excel version 16.61.1 (Microsoft Office 365 for Mac Software, Redmond, WA, USA). FTIR data were deconvoluted using Origin 2019b (OriginLab, Northampton, MA, USA). NMR spectra were analyzed and graphed using MestReNova (Mestrelab Research, Santiago de Compostela, Spain). Semiquantitative particle sizes and circularities were obtained from the electron microscopy images. The dot intensities on cytokine membranes were analyzed using ImageJ (the NIH’s National Institute of Mental Health, Bethesda, MD, USA). All graphing and statistical analyses were conducted using GraphPad Prism 9 (GraphPad Software, Boston, MA, USA). One-way ANOVA and Dunnett’s multiple comparisons were used as statistical tests. Asterisks denote statistical significance, and experimental repeats (*n*) are specified in each figure legend.

## Results and Discussion

Several methods, including emulsification,^24^ complex coacervation,^25^ nanospray drying,^26^ electrospraying,^27^ desolvation,^28^ and self-assembly,^29^ are used to generate protein-based nanoparticles for drug-delivery applications. However, the ability to easily control particle size, achieve yields greater than 80%, and capitalize on biomimetic principles remains an unmet challenge. This study produced silk nanoparticles by antisolvent nanoprecipitation using isopropanol, which requires no complicated procedure or expensive apparatus but instead relies on inducing a self-assembly of the protein to form a core β-sheet-rich nanoparticle with a submicron size. In the presence of alcohol-based solvents, the surface charge disruption and inter- and intramolecular interactions ultimately dehydrate protein molecules to drive particle formation.^30^ The pH, ionic strength, desolvating agent, cross-linking agent, and encapsulated drug content typically govern the size and physicochemical properties of the resulting nanoparticles.^6,21^ This study revealed that the combination of Ca^2+^ and isopropanol maximized yields and modulated and tuned the size and physicochemical properties of the silk nanoparticles. The Ca^2+^ to silk mass ratio used in our study was based on the ratio reported in the silk gland (reviewed in ref (12)). Specifically, the reported ratio was 3000 μg of Ca^2+^ per dried weight of luminal contents (silk = 26% w/w). To adapt this ratio to our silk concentration of 3% w/v, we optimized the Ca^2+^ mass ratio, which resulted in a maximum value of 11.5 mg Ca^2+^ per 1 g silk. The significance of this Ca^2+^ to a silk ratio as well as lower ratios was studied in this work.

### Conformational Changes and Secondary Structure Composition of Aqueous Silk and Silk Nanoparticles

**Figure 1:**
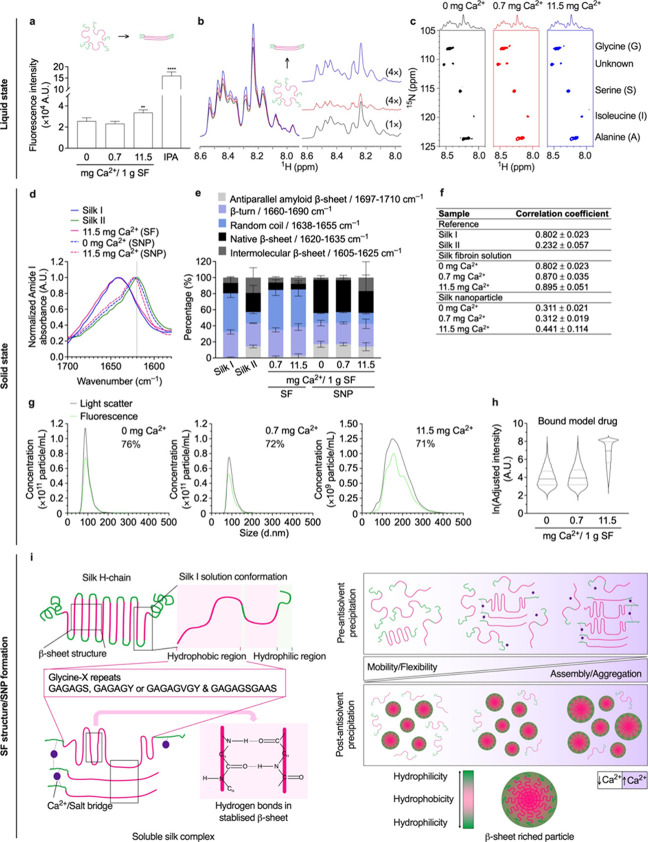
Structural and conformational analyses of silk solutions and nanoparticles. (a) Structural changes of aqueous silk fibroin (SF) in the presence of Ca^2+^ as assessed by thioflavin T assays (ThT); isopropanol (IPA) (*n* = 3), (b) 1D ^1^H NMR, and (c) and 2D [^1^H, ^15^N] HSQC NMR spectra in the NH region (*n* = 1). The 1D ^1^H NMR spectra are shown at the left of the panel; difference spectra (red and blue) compared with control spectrum (black) are shown at the right of panel; all NMR data: black, control; red, with 0.7 mg Ca^2+^ (7% increase); blue, with 11.5 mg Ca^2+^ (17.5% increase). The difference spectra are adjusted with the vertical scale multiplied by a factor of 4. FTIR secondary structure analyses of control silk films with low (silk I) and high (silk II) β-sheet content and silk nanoparticles with (d) the normalized amide I absorbance spectra, (e) secondary structure content, and (f) and correlation coefficient. The percentage of β-sheet content was shown as a summation of β-sheet antiparallel amyloid, β-sheet native, and β-sheet intermolecular structures (*n* = 3). The second-derivative amide I spectrum of an air-dried silk film (amorphous silk denoted silk I) was used as a reference for the correlation coefficient (*R*) calculations (mean ± SD, *n* = 3). (g) The overlay of model drug-loaded silk nanoparticle concentration corresponds to size distribution measured by the nanoparticle-tracking analysis (NTA). The light scatter data are shown as black lines, and the fluorescence data are shown as green lines for 1 mg/mL samples. The percentage is obtained by calculating the area under the curve of the fluorescence data relative to the light scatter data, showing the percentages of model drug-loaded nanoparticles. (h) The fluorescence intensity of the payload detected by nanoparticle tracking. One-way ANOVA and Dunnett’s multiple comparisons test were used for statistical analysis, *p* < 0.05 (*), *p* < 0.01 (**), *p* < 0.001 (***), and *p* < 0.0001 (****). (i) Schematic of the proposed Ca^2+^-enhanced silk self-assembly process. Ca^2+^ increased the yield and enabled particle size tuning, ultimately resulting in β-sheet-rich nanoparticles following antisolvent precipitation. Abbreviations: thioflavin T assays (ThT); isopropanol (IPA); liquid silk fibroin (SF); solid silk nanoparticle (SNP).

The fluorescence thioflavin T assay was used to measure the degree of β-sheet content and the aggregation of silk in the presence of Ca^2+^. The fluorescence intensity of a bound thioflavin T was increased with increasing Ca^2+^ levels ranging from 0.7 to 11.5 mg Ca^2+^ and therefore showed similar characteristics to those of an isopropanol positive control (Figure 1a). The 1D ^1^H NMR spectra revealed no significant differences in chemical shifts in the NH region (8.5–7.8 ppm); however, a consistent trend of increasing peak intensity and altered peak sharpness was observed with an increasing Ca^2+^ mass ratio. The spectra relative to the control (0.0 mg Ca^2+^) for 0.7 and 11.5 mg Ca^2+^ showed an integral increase of 7 and 17.5%, respectively (Figure 1b). The 2D [^1^H, ^15^N] HSQC NMR data showed ^15^N random coil chemical shifts of amino acids that were followed by an alanine residue, including glycine (108.8 ppm), serine (115.7 ppm), isoleucine (119.9 ppm), alanine (123.8 ppm), and an unidentified amino acid (110.8 ppm)^31^ (Figure 1c). The overall ^1^H NMR (10–0 ppm) and ^13^C NMR (190–10 ppm) chemical shift profiles of silk were also elucidated (Figure S2).

The impact of Ca^2+^ on the structural transformation of silk was elucidated by the FTIR analysis to compare the secondary structure content of the silk nanoparticles and precursor materials (i.e., the Ca^2+^-mixed silk solution). The seminal work by Asakura and colleagues, using NMR and other spectroscopic methods, has demonstrated that native silk, prior to spinning, adopts a β-turn structure rather than an α-helix.^32^ This foundational research has informed our FTIR analysis to monitor relative changes (Figure 1e). Additionally, we have included widely accepted peak assignments, which attribute the 1656–1662 cm^–1^ region to the α-helix^33−37^ and relevant variations reported in the literature^20,34,35^ (Figures S3 and S4), enabling cross-study comparisons. While FTIR analysis of silk samples presents challenges, we have supplemented our findings with additional assays, such as NMR and thioflavin T, to strengthen our conclusions. The silk I and silk II references showed a typical amide I region, which shifted from 1640 to 1620 cm^–1^. The spectra of Ca^2+^-mediated and control silk nanoparticles were both consistent with the crystalline silk II reference, indicating an increase in the β-sheet structure (Figure 1d).

The IR fitting of the amide I region was elucidated for the secondary structure content calculation, including the percentages of different β-sheets, β-turns, and random coil structures (Figure S5). The mass ratio did not affect β-sheet structure formation in the Ca^2+^-mixed silk solution phase, which exhibited a low β-sheet content in the 17–18% range. By contrast, the silk nanoparticles showed a β-sheet-enriched content of 56–61%. No significant differences were observed among the silk nanoparticle species in an overall β-sheet content, but an increased proportion of intermolecular β-sheet was observed in the high mass ratio Ca^2+^ (Figure 1e). The correlation coefficient (*R*), which was derived from second derivative amide I spectra and refers to the formulation-induced structural changes of silk nanoparticles compared with air-dried silk films, showed values that ranged from 0.311 ± 0.021 to 0.441 ± 0.114 (Figure 1f).

In the presence of Ca^2+^, the transient salt bridges formed between Ca^2+^ and an estimated 77 carboxylate-substituted amino acids (aspartic acid and glutamic acid) in silk contribute to a strong inter- and intramolecular interaction between silk chains.^38^ These salt bridges stabilize the silk formation network, resulting in increased viscosity and the creation of a gel-like liquid state.^13^ Thus, preincubating a reverse-engineered aqueous silk with Ca^2+^ may cause the formation of a chelating network, thereby yielding a higher order of stabilized β-sheets along with an increase in the Ca^2+^ mass ratio.^12,19^ The Ca^2+^ ions therefore induced a liquid crystalline-like state.^39^ The metal ion valence state has also been reported to affect the self-assembly behavior of silk, whereby Ca^2+^ at 100 positive charges per silk chain can accelerate the formation of a hydrodynamic diameter (*d*_H_) that reduces diffusion and creates a stronger interaction between silk chains.^38^

The antisolvent nanoprecipitation in isopropanol using a semibatch format induced structural changes in which the hydrophilic blocks of the H-chain had a low solubility that promoted the self-assembly of β-sheet-rich silk nanoparticles.^20^ FTIR analysis showed a conformational transition during silk nanoparticle synthesis, as evidenced by the higher β-sheet content of the silk nanoparticles compared to that of the silk I film control. However, in the presence and absence of Ca^2+^, the overall degree of β-sheet content in the respective silk nanoparticles was comparable. This result demonstrates that Ca^2+^ does not significantly affect overall secondary structure changes; however, a high mass ratio Ca^2+^ noticeably increases the proportion of the intermolecular β-sheet structure. We speculate that Ca^2+^ induced a liquid crystalline-like state by influencing silk stability and fluidity.

This study also revealed significant fluorescence intensity of bound thioflavin T in the presence of a high Ca^2+^ mass ratio. Thioflavin T is a fluorescent dye consisting of benzothiazole and dimethylaminobenzene. Changes in rotation of these aromatic rings, derived from the binding or through intercalation, alter the fluorescence emission intensity.^40^ Thioflavin T serves as a gold standard for detecting amyloid binding and formation,^41,42^ β-sheet-rich protein aggregation,^43,44^ and nucleic acid binding and recognition.^40,45^ The thioflavin T findings reported here supported the notion that Ca^2+^ promotes silk self-assembly and increases the β-sheet content in the aqueous phase.

Thioflavin T was also used as a model drug to determine the extent of solvent-accessible beta-sheet structure content, revealing that the percentage of model drug-loaded silk nanoparticles detected by nanoparticle-tracking analysis (NTA) ranged from 71 ± 12 to 76 ± 10% (Figure 1g). The fluorescence intensity measurements showed a similar drug content per particle for the control and low Ca^2+^ mass ratio nanoparticles. However, the production yield was significantly improved, resulting in 360% more particles (Figure S6b). A higher payload intensity was also observed for those silk nanoparticles formed with a 11.5 mg mass ratio of Ca^2+^ per 1 g of silk fibroin (Figure 1h, Figure S6a). Therefore, Ca^2+^ not only increased particle yield and size (detailed below) but also significantly increased drug loading (Figure S6a). We hypothesize that Ca^2+^ may enhance both silk–silk interaction and payload–silk interaction, resulting in a higher payload content in silk nanoparticles at increased Ca^2+^ mass ratios in parallel with size tuning.

The conformational changes in silk were further explored by 1D ^1^H NMR and 2D [^1^H, ^15^N] HSQC NMR analysis. Notable increases in signal area and intensity were recorded for the NH resonance region of the 1D ^1^H NMR data as the Ca^2+^ content increased. This NMR signal increase implies that a greater proportion of all the labile hydrogens occupied the same local NH chemical environment, indicating an alteration of protein stability (i.e., denaturation, fragmentation, and self-assembly).^46^ Our study showed that increasing the Ca^2+^ concentration increased the area of the NH region, suggesting a change in the on–off exchange rate of the labile NH protons. If we consider that the increase in signal correlates with a reduced rate of exchange, then the NH protons spend more of their time attached to nitrogen. One implication of this could be a greater degree of a liquid crystalline-like state, which could subsequently aid silk nanoparticle formation.

Our NMR studies enabled us to monitor the molecular mobility, and our data suggested a greater degree of self-assembly at the onset of the assembly into larger particles.^46,47^ This hypothesis is supported by earlier evidence showing that metal ions influenced the folding characteristics of silk fibroin, as indicated by an increase in the ^13^C CP-MAS NMR spectra intensity in the alanyl C_β_ region (22–12 ppm) and the transitioning of the silk from an α-helix to a β-sheet conformation.^48^ The most abundant amino acids in the silk heavy chain are glycine, alanine, and serine, and our 2D [^1^H, ^15^N] HSQC solution NMR data showed that their respective signals displayed random coil chemical shifts that are consistent with a mobile, “unstructured” silk molecule (i.e., no β-sheets), although these hydrophobic amino acid motives are ultimately implicated in β-sheet formation and silk mechanics.^7,11^

Silk nanoparticle formation occurs when silk is combined with an antisolvent, resulting in supersaturation and the nucleation of particles through the Gibbs free-energy self-assembly mechanism.^49^ During this process, collisions between silk chains form complexes that develop into stable nuclei, and as protein concentration decreases, growth in larger nuclei is enabled.^20,49^ Overall, our findings confirmed the impact of Ca^2+^ on the silk conformation and stability. This effect arose due to the formation of salt bridges between Ca^2+^ and acidic amino acids that then caused a strong attractive force between silk protein chains,^12,13,19,32^ reduced the number of unfolded silk chains, and yielded silk soluble complexes. This phenomenon enhanced silk folding and a conformational transition that favored β-sheet structure^14,48^ and created a better hydrogen bonding geometry of C=O groups and/or NH groups in the β-sheet conformation.^50^ Therefore, we speculate that the local environment of the key amino acid residues was more stabilized. These stabilized silk complexes enabled the silk assembly in isopropanol to proceed because the overall entropy in the system was reduced through Ca^2+^-mediated preordering of the silk structure into a liquid crystalline-like state. This, in turn, reduced the “activation barrier” to silk nanoparticle formation, making it “easier” for silk nanoparticles to grow (and for gel formation to occur more readily) (Figure 1i). The high Ca^2+^ mass ratio resulted in a significant difference in the bound thioflavin T fluorescence intensity and a dramatic difference in ^1^H NMR spectra. Taken together, our findings confirmed that Ca^2+^ significantly impacts the silk nanoparticle size-tuning process in a Ca^2+^ concentration-dependent manner.

### Particle Size and Morphology of Silk Nanoparticles

**Figure 2:**
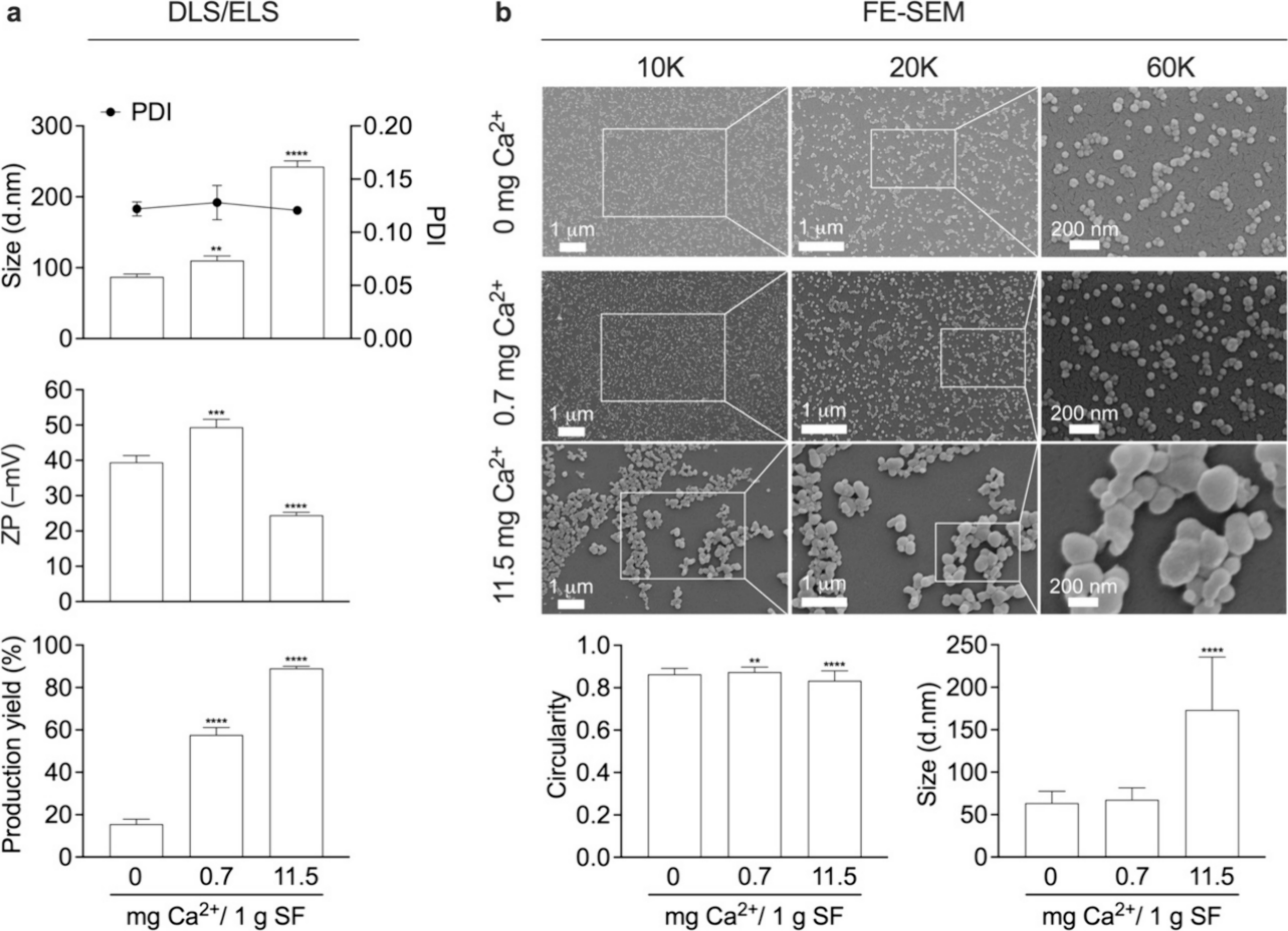
Silk nanoparticle characteristics. (a) Analysis of size (dynamic light scattering, DLS; polydispersity index, PDI), zeta potential (ZP) (electrophoretic light scattering, ELS), and production yield of silk nanoparticles (*n* = 3). (b) Field emission scanning electron microscopy (FE-SEM) images were reported at 10, 20, and 60K magnifications. 400–500 particles and 100–150 particles derived from at least three different regions of interest were used to perform size and circularity calculations, respectively. Metal ion ratios were expressed as mg per 1 g of silk fibroin (SF). One-way ANOVA and Dunnett’s multiple comparison test were used for statistical analysis; *p* < 0.05 (*), *p* < 0.01 (**), *p* < 0.001 (***), and *p* < 0.0001 (****). PDI: polydispersity index.

Silk nanoparticles were manufactured at a low (0.7 mg of Ca^2+^) and a high Ca^2+^ (11.5 mg of Ca^2+^) mass ratio to mimic the natural Ca^2+^ mass ratio per gram of silk fibroin reported in the middle silk gland as the highest experimental ratio. The impact of Ca^2+^ on silk nanoparticle physicochemical properties was determined by using dynamic and electrophoretic light scattering. The increase in Ca^2+^ mass ratio significantly increased the particle size from 87.1 to 110.3 and 262.8 d.nm for low and high mass ratio Ca^2+^, respectively. However, the samples maintained a low polydispersity index. The production yield was significantly increased from 16 to 89% in a Ca^2+^ concentration-dependent manner (Figure 2a). We demonstrate here that the particle size increased with rising Ca^2+^ concentrations. Our findings are supported by an orthogonal study by Lee and co-workers, who showed that Ca^2+^ concentrations influenced the extent of silk-dextran liquid–liquid phase separation, thereby affecting coacervate size and the turbidity of the solutions.^51^ The corresponding phase separation map^51^ of silk-dextran in response to Ca^2+^ ion concentration showed a one-phase and two-phase system at the corresponding low and high Ca^2+^ ion concentrations used in the present work. However, the absence of a crowding agent in our study negates the liquid–liquid phase separation phenomenon.

We used field emission scanning electron microscopy to assess the morphology and size of the dehydrated silk nanoparticles. All silk nanoparticles had a spherical morphology with coarse surface appearance at low Ca^2+^ mass ratios, but the surfaces became smooth at high Ca^2+^ mass ratios and a circularity of 0.8–0.9 was maintained. These images showed a consistent trend in particle size changes compared with the dynamic light scattering, increasing in size from 64 (±14) to 173 (±62) nm (Figure 2b).

Morphological assessment using electron microscopy confirmed the spherical topology of the silk nanoparticles, but the size was smaller compared with dynamic light scattering measurements (Figure 2). This discrepancy can be expected due to different analysis modes and sample states (i.e., dried versus hydrated). Corroborating this, larger particles showed a greater extent of shrinkage (27–40%) that in turn implies greater swelling capacity in solution.

The zeta potential value reflects the nanoparticle surface electrical charge, whereby a higher zeta potential indicates greater stability and less aggregation.^52^ Ca^2+^ affected the zeta potential value of the silk nanoparticles, as a low mass ratio of Ca^2+^ raised the zeta potential value from −39.5 to −49.4 mV, while a high mass ratio of Ca^2+^ dropped the zeta potential value to −24.4 mV (Figure 2a). Increasing the Ca^2+^ mass ratio was also equivalent to increasing the concentration of counterion (Cl^–^) in a desolvating system. This possibly changes the ionic balance of the silk nanoparticles and affects the net charge loading of the particles. This would lead to a higher zeta potential for a low mass ratio of Ca^2+^ but a lower zeta potential for a high mass ratio of Ca^2+^. The high Ca^2+^ mass ratio might indicate a saturation of ion-mediated electrostatic shielding to a specific charge interaction of both Ca^2+^ and Cl^–^ on silk protein.^53^ Due to the dominant forces in protein folding, especially a hydrophobic interaction occurs during a silk assembly and silk nanoparticle formation, the polar/charged residues will be exposed on a silk nanoparticle surface protecting a rigid cored structure (β-sheet).^53^ This changed the net charge surface of silk nanoparticles, resulting in a decreased zeta potential index. By contrast, no change was observed in the polydispersity index, indicating a dispersion appropriate for nanomedicines (polydispersity index < 0.2).^54,55^ The proposed physicochemical properties and production yield of the resulting silk nanoparticles align with the characteristics of nanoparticles intended for drug delivery (detailed in Table S1).

### Cellular Responses to Silk Nanoparticles

**Figure 3:**
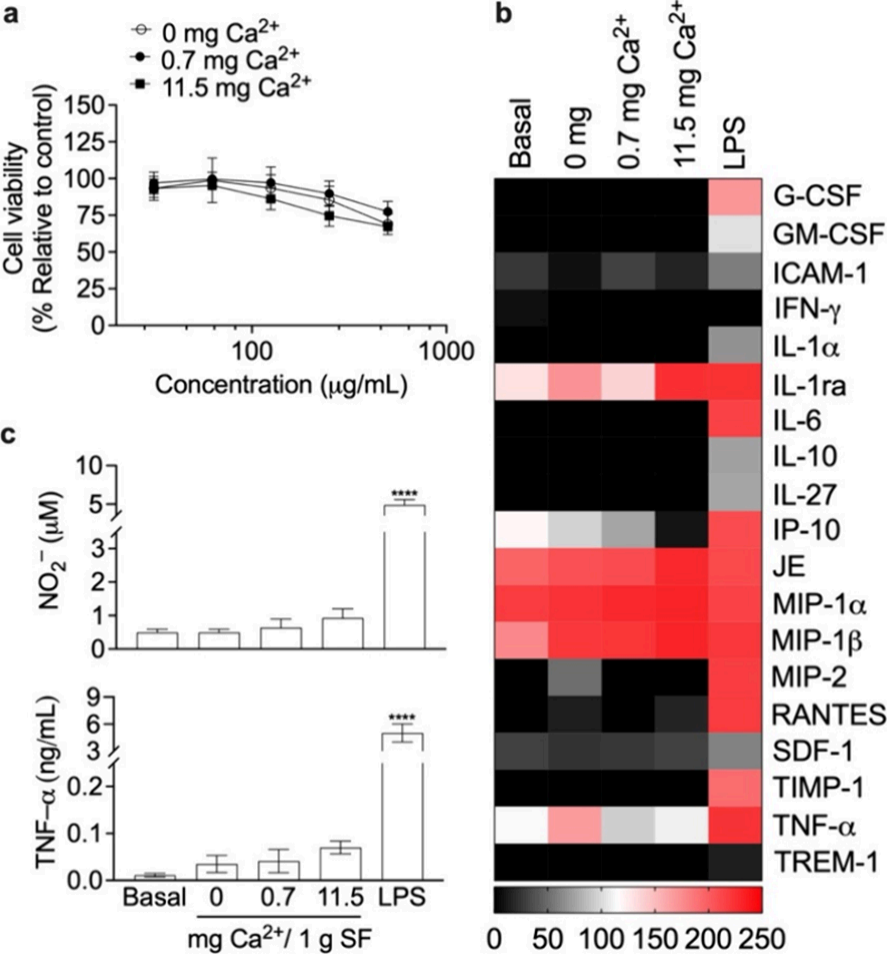
Impact of silk nanoparticles on macrophages. (a) Cell viability and inflammatory responses, (b) inflammatory cytokine expression level (scale bar pixel intensity), and (c) endogenous nitrite (NO_2_^–^) and TNF-α levels in response to the silk nanoparticles. Macrophages treated with complete media supplemented with 200 ng/mL lipopolysaccharide (LPS) served as an inflammation-positive control. SF: Silk fibroin. One-way ANOVA and Dunnett’s multiple comparisons test were used for statistical analysis, *p* < 0.05 (*), *p* < 0.01 (**), *p* < 0.001 (***), and *p* < 0.0001 (****) (*n* = 3).

The biocompatibility of silk nanoparticles was tested using RAW 264.7 murine macrophage cells. The low Ca^2+^ mass ratio-mixed silk nanoparticles exhibited a low cytotoxicity comparable to that observed for control silk nanoparticles, with a growth reduction of <15% at a silk nanoparticle concentration of <250 μg/mL. Similarly, silk nanoparticles applied at 31.25–125 μg/mL caused a growth reduction of <20%. Cell viability gradually decreased over the test concentration range; however, the IC50 remained higher than the maximum concentration (500 μg/mL; Figure 3a).

Inflammatory responses to silk nanoparticles were elucidated by comparison to lipopolysaccharide-treated macrophages, which served as a positive control for inflammation. Inflammatory cytokines and chemokines in the cell culture media were measured after 24 h of nanoparticle exposure. The Ca^2+^-mediated silk nanoparticle noticeably upregulated interleukin-1 receptor antagonist (IL-1ra), monocyte chemoattractant protein-1 (MCP-1/CCL2/JE), macrophage inflammatory protein-1α (MIP-1α), and macrophage inflammatory protein-1β (MIP-1β), whereas interferon gamma-induced protein 10 (IP-10) was downregulated (Figure 3b and Figure S7). All tested silk nanoparticles induced negligible amounts of endogenous nitrite (NO_2_^–^) when compared with the positive control (4.9 mM). The TNF-α levels (<0.07 ng/mL) showed no significant differences when comparing baseline and silk nanoparticle groups (Figure 3c).

In this study, the cytotoxicity and inflammatory response following silk nanoparticle exposure were evaluated in an experimental macrophage model to profile nanoparticle biocompatibility. In therapeutic drug-delivery systems, nanoparticles will be exposed to circulating biomolecules in a systemic circulation or at the administration site. Monitoring macrophage health, to gauge biocompatibility, is useful because macrophages serve as a key first-line defense mechanism.^56,57^ Nanoparticles have recently been implicated in alterations of the macrophage phenotype, including induction of polarization and reprogramming and modulation of pro- and anti-inflammatory cytokines and chemokines.^58−60^ However, treatment of macrophages with any of the tested silk nanoparticles resulted in no significant upregulation of endogenous NO_2_^–^ and TNF-α.

Particle size-dependent cellular responses are described in the literature, for example, a greater degree of inflammation was induced by submicron amorphous silica particles (30–1000 d.nm) than by particles >1000 nm,^61^ while a higher level of cytotoxicity, inflammation, genotoxicity, and developmental toxicity was promoted by small silver nanoparticles (20 d.nm) than by larger particles (80 and 113 d.nm).^62^ The effects of any silk nanoparticles on macrophages revealed negligible variations in JE or MIP-1α, an upregulation of IL-1ra and MIP-1β, and a downregulation of IP-10 compared to baseline levels. Here, none of the tested silk nanoparticles promoted an excessive production of proinflammatory cytokines (IL-1α, IL-6, G-CSF, GM-CSF, and TIMP-1),^63−65^ an anti-inflammatory cytokine (IL-10),^66^ or a proinflammatory chemokine (TREM-1)^67^ compared with lipopolysaccharide-activated macrophages.

## Conclusions

This study used Ca^2+^ ion spiking of liquid silk to coordinate silk solution properties and to promote silk nanoparticle self-assembly using antisolvent nanoprecipitation in a semibatch format. Successful improvement of the physicochemical properties and scalability of silk nanoparticles was achieved by mimicking the natural function of Ca^2+^ in silk processing by silkworms. The Ca^2+^:silk mass ratio of storage silk in the middle silk gland was selected as our high Ca^2+^ condition and was compared to a 16-fold lower condition (0.7 mg/g). The findings confirmed the effect of Ca^2+^ on silk conformational and structural properties. In the presence of Ca^2+^, we observed the formation of a liquid-crystalline-like state that transitioned to solid β-sheet-rich silk nanoparticles. These nanoparticles showed good biocompatibility in our macrophage model.

## Data Availability

All data created during this research are openly available from the University of Strathclyde-Pure, at https://doi.org/10.15129/88da6654-2b38-432f-b5c1-7693ca101645.

## Supporting Information Available

Schematic of aqueous silk fibroin preparation and silk nanoparticle manufacture (Figure S1); NMR spectra of silk fibroin (Figure S2); the exemplary IR fitting of the amide I region, including the peak assignment for α-helix, was performed for Ca^2+^-mixed silk fibroin (0.7 and 11.5 mg Ca^2+^ SF) and Ca^2+^-mixed silk nanoparticles (0, 0.7, and 11.5 mg Ca^2+^ SNP), in comparison with freeze-dried silk powder (silk I) and EtOH-treated silk film (silk II) controls (Figure S3); secondary structure content (Figure S4); the exemplary IR fitting of the amide I region for Ca^2+^-mixed silk fibroin (0.7 and 11.5 mg Ca^2+^ SF) and Ca^2+^-mixed silk nanoparticle (0, 0.7, and 11.5 mg Ca^2+^ SNP) in comparison with the freeze-dried silk powder (silk I) and EtOH-treated silk film (silk II) control (Figure S5); thioflavin T-loaded silk nanoparticles (ThT-loaded SNP) manufactured in the 0–11.5 mg Ca^2+^ range (Figure S6); the proposed SNP physicochemical properties and production yield (Table S1); inflammatory cytokine detection in RAW 264.7 murine macrophage in response to silk nanoparticle (Figure S7) (PDF)

## Abbreviations

*B. mori*: *Bombyx mori*
Ca^2+^: calcium ion
DLS: dynamic light scattering
DMEM: Dulbecco’s modified eagle medium
FTIR: Fourier transform infrared
G-CSF: granulocyte colony stimulating factor
GM-CSF: granulocyte-macrophage colony-stimulating factor
HSQC: heteronuclear single quantum correlation
IL-1α: interleukin-1α
IL-1ra: interleukin-1 receptor antagonist
IL-6: interleukin-6
IL-10: interleukin-10
IP-10: interferon gamma-induced protein 10
CCL2, JE, MCP-1: monocyte chemoattractant protein-1
IPA: isopropanol
MIP-1α: macrophage inflammatory protein-1α
MIP-1β: macrophage inflammatory protein-1β
MIP-2: macrophage inflammatory protein-2
NMR: nuclear magnetic resonance
NO_2_^–^: nitrite
PDI: polydispersity index
RANTES: regulated upon activation, normal T cell expressed and presumably secreted
SF: silk fibroin
SNP: silk nanoparticle
ThT: thioflavin T
TIMP-1: tissue inhibitor matrix metalloproteinase 1
TNF-α: tumor necrosis factor alpha
TREM-1: triggering receptor expressed on myeloid cell 1
ZP: zeta potential
