# Supplementary material for: Biomimetic Silk Nanoparticle Manufacture: Calcium Ion-Mediated Assembly
Source: ACS Biomater Sci Eng. 2025 Jan 30;11(3):1847–56. doi: 10.1021/acsbiomaterials.4c02175 (PMC11897946; doi:10.1021/acsbiomaterials.4c02175)
Supplement: Supplementary file 1 — ab4c02175_si_001.pdf [file ab4c02175_si_001.pdf]

Biomimetic silk nanoparticle manufacture: calcium ion-mediated assembly

Napaporn Roamcharern[a]*, Saphia A. L. Matthew*[a]*, Daniel J. Brady*[b]*, John A. Parkinson*[c]*, Zahra Rattray*[a*]*, F. Philipp Seib*[a,b,d*]

[a]*Strathclyde Institute of Pharmacy and Biomedical Sciences, University of Strathclyde, 161 Cathedral St., Glasgow G4 0RE, Scotland, UK*

[b]*Fraunhofer Institute for Molecular Biology and Applied Ecology, Branch Bioresources, Ohlebergsweg 12, 35392 Giessen, Germany*

[c]*Department of Pure and Applied Chemistry, University of Strathclyde, 295 Cathedral Street, Glasgow G1 1XL, Scotland, UK*

[d]*Friedrich Schiller University Jena, Institute of Pharmacy, Department of Pharmaceutics and Biopharmaceutics, Lessingstr. 8, 07743 Jena, Germany*

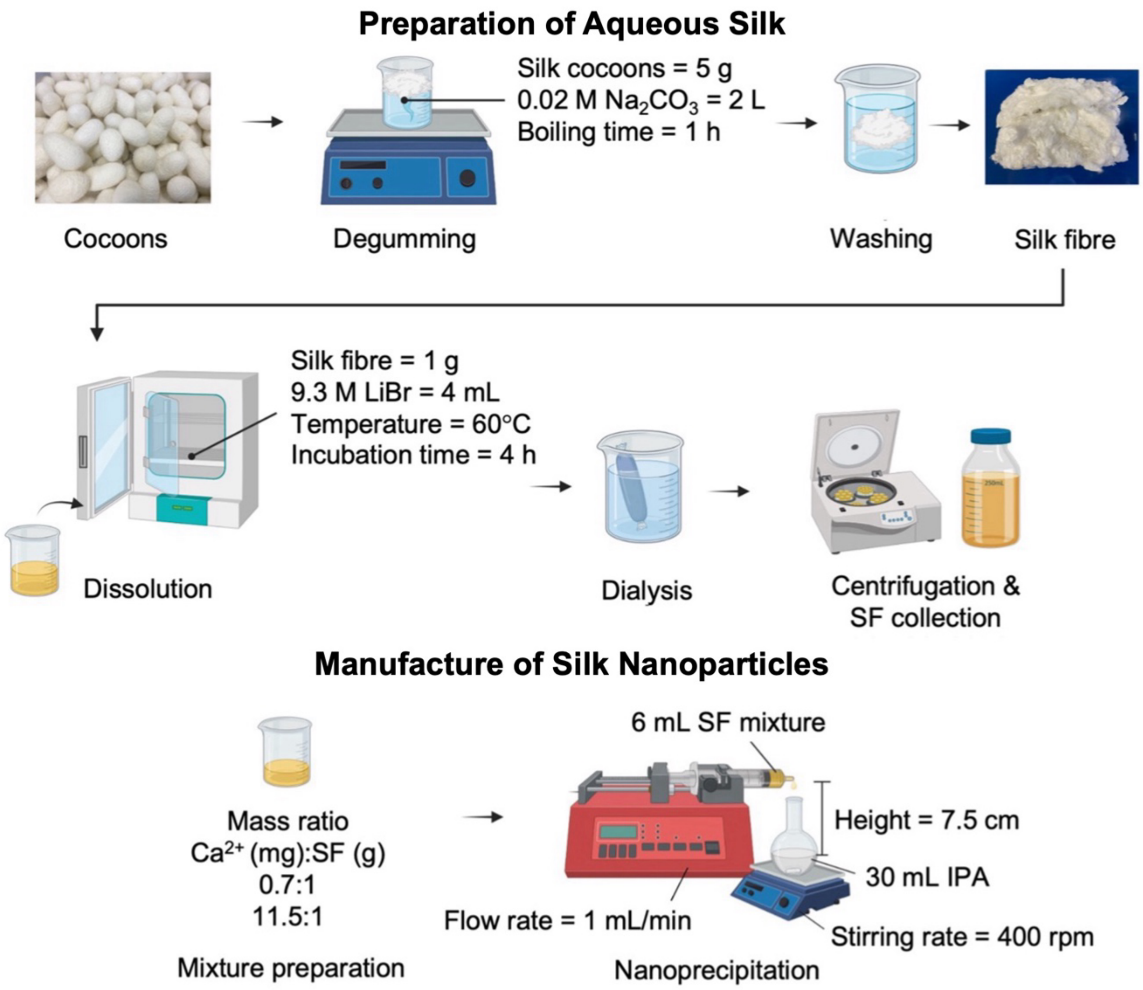

Figure S1. Schematic of aqueous silk fibroin preparation and silk nanoparticle manufacture. Created in BioRender. Rattray, Z. (2025) https://BioRender.com/ b65b962

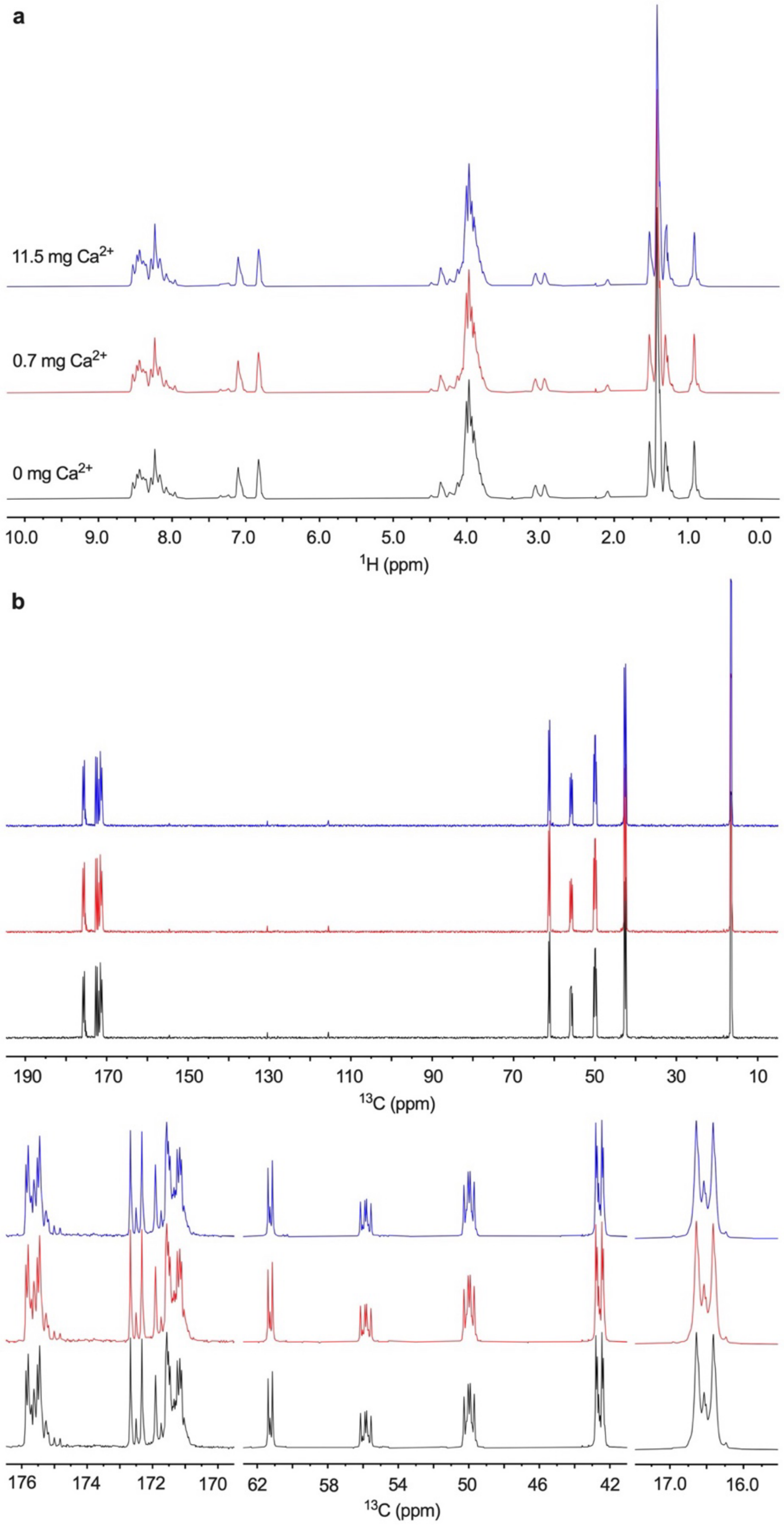

Figure S2. NMR spectra of silk fibroin. (a) 1D ^{1}H-NMR and (b) 1D ^{13}C-NMR spectra; all NMR data: black - control; red - with 0.7 mg Ca^{2+}; blue - with 11.5 mg Ca^{2+} (n = 1).

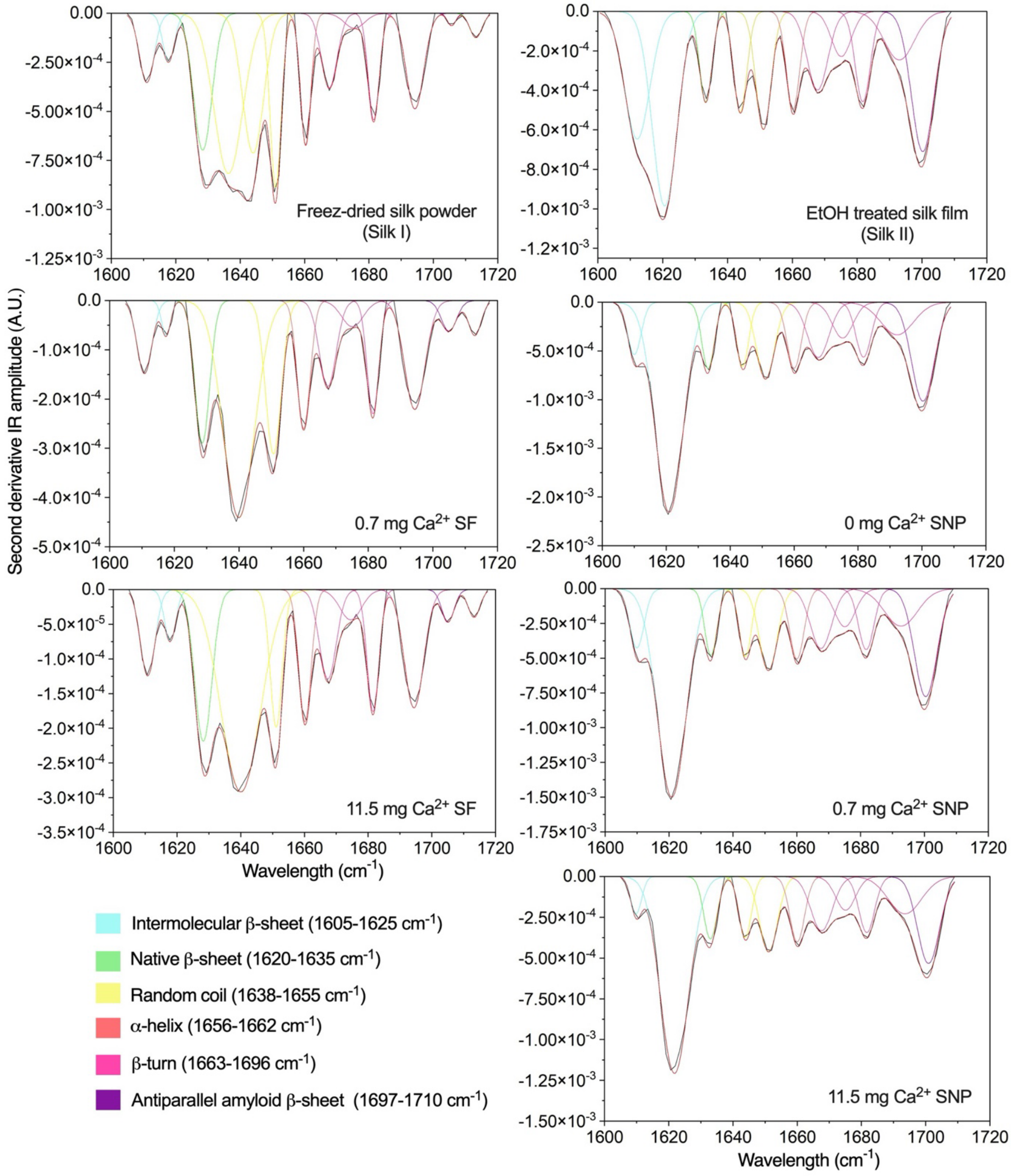

Figure S3. The exemplary IR fitting of the amide I region, including the peak assignment for α-helix, was performed for Ca^{2+}-mixed silk fibroin (0.7 and 11.5 mg Ca^{2+} SF) and Ca^{2+}-mixed silk nanoparticles (SNP) (0, 0.7, and 11.5 mg Ca^{2+} SNP), in comparison with freeze-dried silk powder (silk I) and EtOH-treated silk film (silk II) controls. The freeze-dried silk powder (silk I) serves as the 0 mg Ca^{2+}-mixed silk (n = 1).

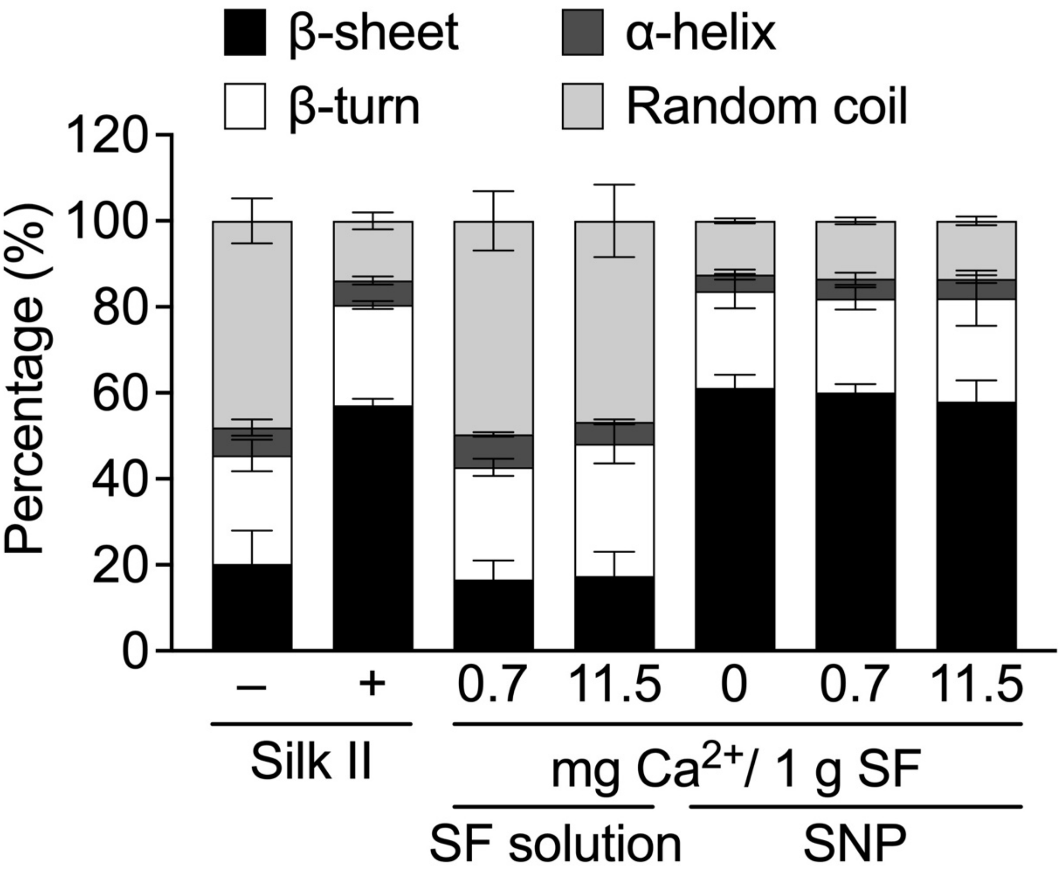

Figure S4. Secondary structure content. The percentage of β-sheet content was shown as a summation of β-sheet antiparallel amyloid, β-sheet native, and β-sheet intermolecular structures (n = 3).

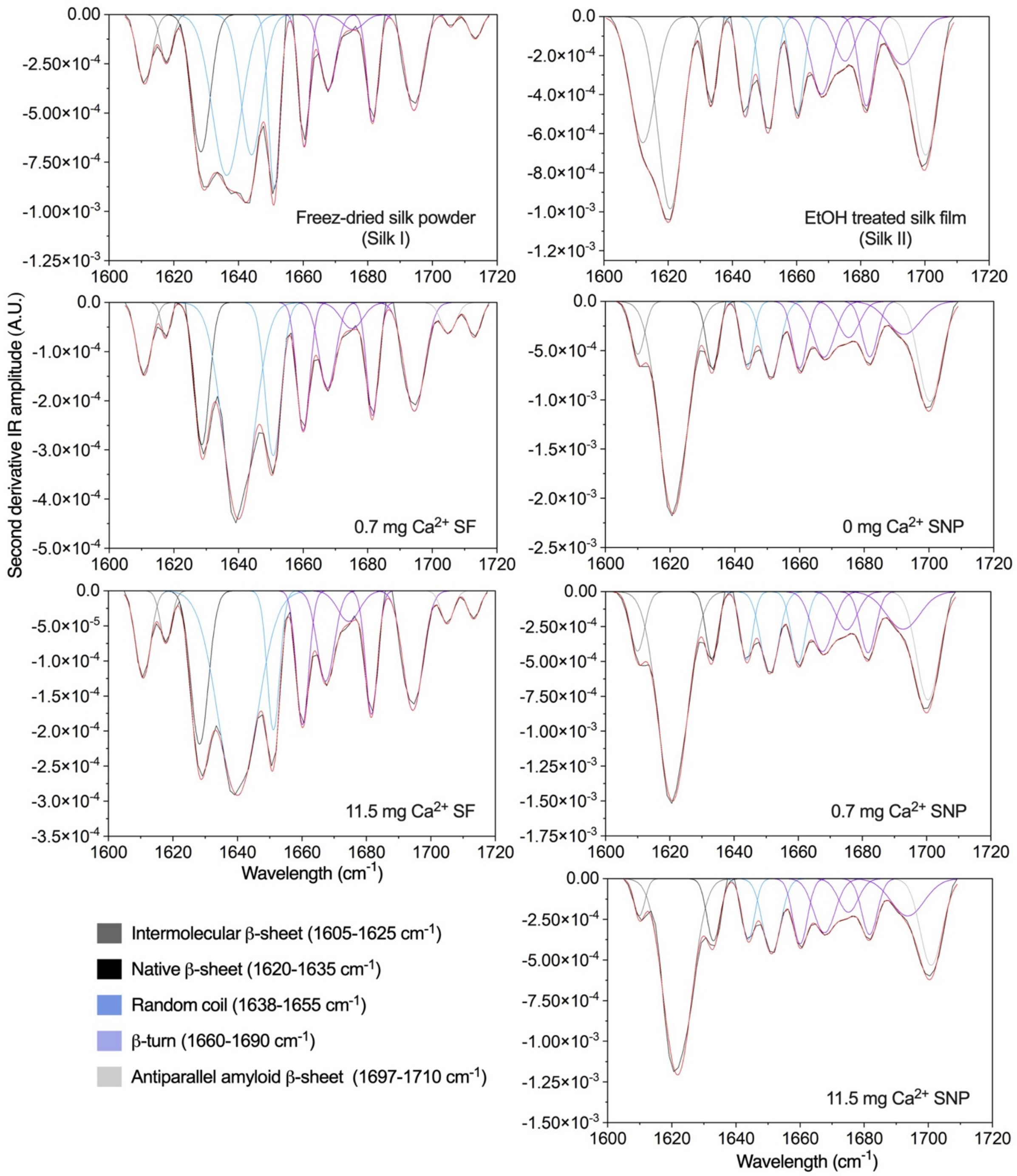

Figure S5. The exemplary IR fitting of the amide I region for Ca^{2+}-mixed silk fibroin (0.7 and 11.5 mg Ca^{2+} SF) and Ca^{2+}-mixed silk nanoparticles (0, 0.7 and 11.5 mg Ca^{2+} SNP) in comparison with the freeze-dried silk powder (silk I) and EtOH-treated silk film (silk II) control. The freeze-dried silk powder (silk I) serves as a 0 mg Ca^{2+}-mixed silk (n =1).

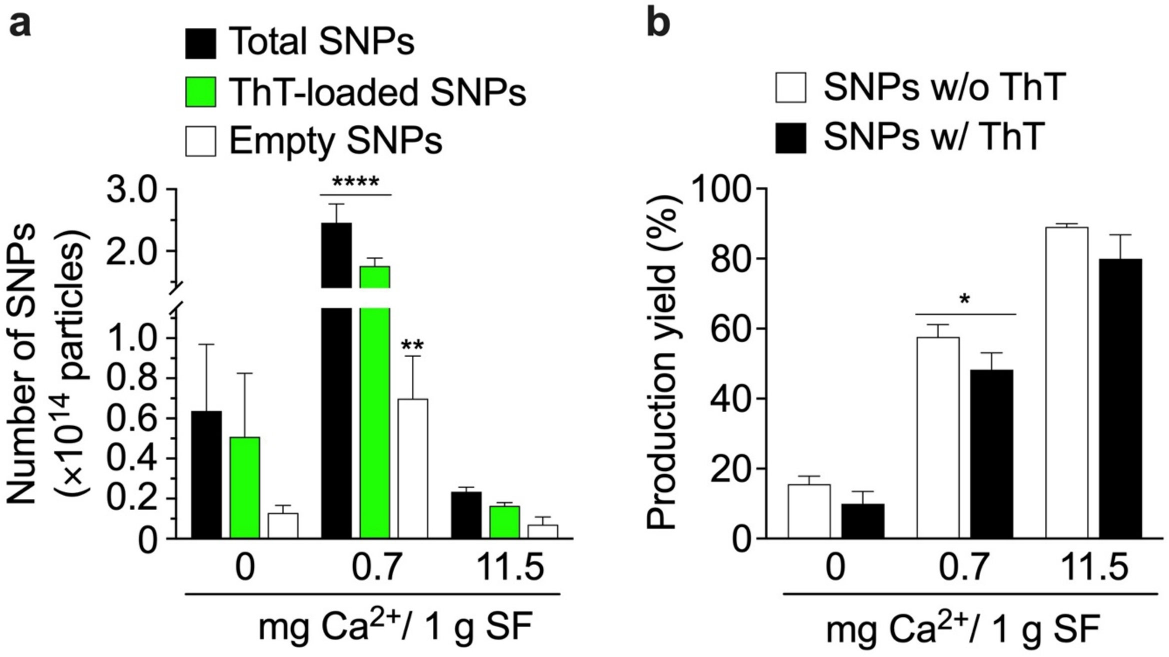

Figure S6. Thioflavin T-loaded silk nanoparticles (ThT-loaded SNP) manufactured in the 0-11.5 mg Ca2+ range. (**a**) The fraction of ThT-loaded and empty silk nanoparticles was elucidated by nanoparticle tracking analysis with a subtraction between light scattering and fluorescent data. (**b**) The production yield of silk nanoparticles obtained with and without ThT addition was calculated and normalized to dried silk fibroin (SF) weight. The Two-way ANOVA and Šídák's multiple comparisons test were used for statistical analysis, $p < 0.05$ (*), $p < 0.01$ (**), $p < 0.001$ (***), and $p < 0.0001$ (****) ($n = 3$).

Table S1. The proposed silk nanoparticle (SNP) physicochemical properties and production yield.

Parameter	Referenced SNP[20]	Proposed SNP
Particle size (d.nm)	114	100–500 [68]
Polydispersity index (PDI)	0.14	≤0.2 [69, 55]
Zeta potential (mV)	–33	±30 or higher [70, 71]
Production yield (%)	9	>20

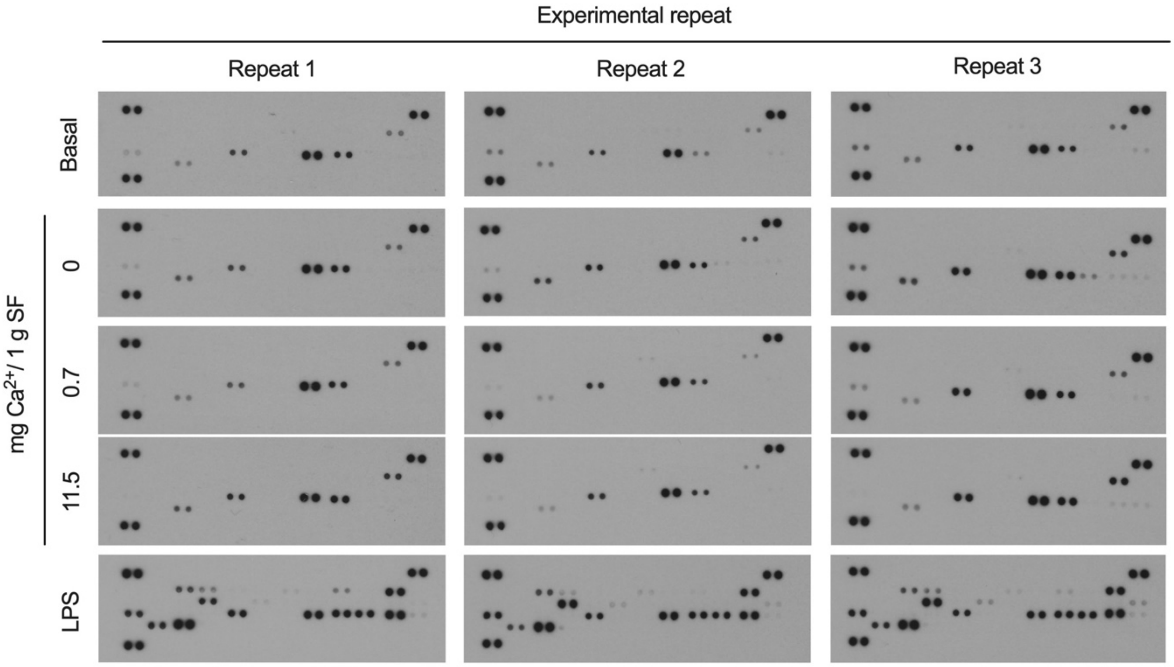

Figure S7. Inflammatory cytokine detection in RAW 264.7 murine macrophage in response to silk nanoparticle. Expressed cytokines obtained from three experimental repeats. Macrophages treated with complete media supplemented with 200 ng/mL lipopolysaccharide served as the positive control. The cytokines and chemokines were processed according to the manufacturer's instructions.

References

(68) Mok, Z. H. The effect of particle size on drug bioavailability in various parts of the body. *Pharmaceutical Science Advances*. 2023, 100031.

(69) Çapan, Y.; Sahin, A.; Tonbul, H. Drug delivery with targeted nanoparticles: in vitro and in vivo evaluation methods. *CRC Press*. 2021.

(70) Clogston, J. D.; Patri, A. K. Zeta potential measurement. Characterization of nanoparticles intended for drug delivery. 2011, 63-70.

(71) Öztürk, K.; Kaplan, M.; Çalış, S. Effects of nanoparticle size, shape, and zeta potential on drug delivery. *International Journal of Pharmaceutics*. 2024, 124799.
